# Predictive Value for Preeclampsia of sFlt‐1:PlGF Ratio in Pregnancy Complicated by Gestational Diabetes

**DOI:** 10.1002/dmrr.70217

**Published:** 2026-08-20

**Authors:** Fabrizia Citro, Cristina Bianchi, Francesca Nicolì, Michele Aragona, Giovanni De Gennaro, Stefano Del Prato, Giuseppe Penno, Alessandra Bertolotto, Lorella Battini

**Affiliations:** ^1^ Department of Clinical and Experimental Medicine University of Pisa Pisa Italy; ^2^ Department of Endocrine‐Metabolic and Transplant Surgery and Medicine University Hospital of Pisa Pisa Italy; ^3^ Endocrine Diseases and Regional Diabetes Mellitus Center ASST Bergamo Ovest Treviglio Italy; ^4^ Metabolic Diseases and Diabetes Unit Misericordia Hospital Grosseto Italy; ^5^ Interdisciplinary Research Center ‘Health Science,’ Sant'Anna School of Advanced Studies Pisa Italy; ^6^ Maternal‐Infant Department University Hospital of Pisa Pisa Italy

**Keywords:** gestational diabetes, hypertensive disorders, placental angiogenic factors, predictive risk factors, preeclampsia, pregnancy, sFlt1/PlGF ratio

## Abstract

**Background and Aims:**

Gestational diabetes (GDM) and preeclampsia (PE) are major complications of pregnancy. The ratio of soluble Fms‐like tyrosine kinase 1 to placental growth factor (sFlt‐1/PlGF) may predict the short‐term absence of PE in pregnant women with clinically suspected PE. The aim of this study was to determine the role of placental angiogenic factors in predicting PE risk in women with pregnancies complicated by GDM.

**Materials and Methods:**

In this prospective observational study, pregnant women were enrolled at the time of screening for GDM. sFlt‐1 and PlGF were measured at 24–28, 28–32 and 35–37 weeks of gestation. Univariate and multivariate analysis were used to investigate risk factors for PE.

**Results:**

Of 398 women, 213 (53.5%) had GDM and 18 (5%) developed PE (67% with GDM). GDM women developing PE had significantly lower levels of PlGF at 24–28, 28–32 and 35–37 gestational weeks, and higher sFlt1 and sFlt1/PlGF at 28–32 and 35–37 gestational weeks compared with GDM women without PE. On a multivariate analysis, PlGF and sFlt1/PlGF were associated with PE, regardless of other clinical data. A sFlt1/PlGF ratio > 38 at 28–32 gestational weeks identified GDM women at risk of developing PE within the ensuing 4 weeks, with 100% sensitivity and good specificity.

**Conclusion:**

In women with GDM, the sFlt1/PlGF ratio can identify those at higher risk of PE.

**Trial Registration:**

NCT04877119: 2021‐04‐29

## 1 Introduction

Preeclampsia (PE) is a multisystem, placentally‐mediated hypertensive disorder, affecting 2%–8% of pregnancies worldwide [1]. PE is defined as the appearance, starting from 20 gestational weeks, of hypertension associated with at least one typical clinical‐biochemical signor symptoms such as proteinuria, acute kidney injury, hepatic and/or neurological involvement, utero‐placental dysfunction (i.e., altered Doppler velocimetry of the uterine and umbilical arteries, Intrauterine Growth Restriction (IUGR) or intrauterine foetal death), thrombocytopoenia, haemolysis, or disseminated intravascular coagulation [2].

Although the cause of PE is not fully understood yet, placental malperfusion due to abnormal remodelling of maternal spiral arteries, may play a central role [3]. Accordingly, placental angiogenic factors have been identified as predicting biomarkers of PE, with the main emphasis on the pro‐angiogenic Placental Growth Factor (PlGF) and the anti‐angiogenic soluble Fms‐like tyrosine kinase‐1 (sFlt‐1). Lower levels of PlGF and an increased sFlt‐1 to PlGF ratio in the first and second trimesters of pregnancy have been shown to precede the clinical onset of PE [4, 5, 6]. In the PROGNOSIS study, sFlt‐1/PlGF ≤ 38 in women at 20 through 35 weeks of gestation was found to predict short‐term absence of PE in women in whom the syndrome was clinically suspected [7].

Epidemiological factors have also been associated with increased risk of PE, such as nulliparity, advanced maternal age, personal or family history of PE in previous pregnancies, and multiple pregnancies. Moreover, medical conditions like chronic hypertension, obesity, kidney disease, hypercoagulability conditions, and diabetes are all at increased risk of PE [6]. Many studies have described a higher rate of PE in women with pre‐gestational and gestational (GDM) diabetes, suggesting a role for hyperglycemia in promoting placental vascular endothelial dysfunction, through Advanced Glycation End Products (AGEs) formation, oxidative stress, and inflammation [8, 9, 10, 11]. Finally, GDM and PE share common risk factors, such as advanced age and obesity, that may account for the higher rate of PE in these women [12, 13]. However, whether the sFlt1/PlGF ratio is also predictive of the short‐term risk of PE in women with GDM or at high risk for GDM has not been established. Therefore, the aim of this study was to assess the role of placental angiogenic factors from the time of the screening for GDM in predicting PE risk and to assess whether the cut‐off value of 38 for the sFlt1/PlGF ratio is also effective in these women [6].

## 2 Materials and Methods

### 2.1 Subjects

The PREDICTION (PREeclampsia in DIabetiC gestation) study (identification code on clinicaltrial.gov: NCT04877119), prospectively enrolled pregnant women who performed a 75‐g Oral Glucose Tolerance Test (OGTT) for the screening of GDM from October 2019 to June 2022, at the Diabetes Section of the University Hospital of Pisa. Pregestational diabetes, pregestational hypertension, chronic kidney disease, twin pregnancy, or PE already diagnosed in the current pregnancy were considered exclusion criteria. The study complies with the Declaration of Helsinki and was approved by the local Ethical Committee. All women provided a written informed consent for study participation and personal data treatment prior to enrollment.

### 2.2 Study Design

As previously described [14], women were enrolled at the time of 75‐gr OGTT for GDM screening when clinical, anthropometric, metabolic data and risk factors for GDM were also collected. A 75‐g OGTT was performed between 16–18 or 24–28 gestational weeks, based on pre‐pregnancy risk factors as indicated by the Italian guidelines [15]. GDM was defined according to the IADPSG/WHO 2013 criteria [16, 17]. Women with GDM were seen at 2–3 weeks intervals for glycemic profiles, weight gain and blood pressure monitoring, while HbA1c and lipid profile were measured at 28–32 gestational weeks. An additional blood sample for the measurement of placental angiogenic factors (PlGF, sFlt1 and sFlt1/PlGF ratio) was obtained in all women at the time of OGTT screening, as well as at 28–32 and 35–37 gestational weeks in women with GDM diagnosis. Samples were collected in tubes containing K2 EDTA anticoagulant + gel and assays were performed at the Laboratory of University Hospital of Pisa using an ELISA technique (Elecsys, Roche Diagnostics, Italy).

PE was diagnosed according to national [18] and international [19, 20] guidelines. Diagnostic criteria were: onset of proteinuria (urine dipstick ≥ 2+, protein in 24‐h urine collection ≥ 300 mg, protein in urine sample ≥ 30 mg/dL, protein/creatinine ratio in urine sample ≥ 30 mg/mmol) and gestational hypertension (systolic blood pressure ≥ 140 mmHg and/or diastolic blood pressure ≥ 90 mmHg in two different occasions at least 20 minutes apart) after 20 gestational weeks.

### 2.3 Statistical Analysis

Data analysis was performed using the *IBM SPSS Statistics V.20* statistical analysis programme. Continuous variables were expressed as mean ± Standard Deviation (SD), if normally distributed, and as median (interquartile range; IQR) if not; categorical variables were expressed as percentages. Normality was checked using the Kolmogorov–Smirnov test. Student's *t*‐test and Mann–Whitney test were used to compare continuous variables as appropriate. Univariate and multivariate analysis were used to investigate risk factors for PE. Receiver operating characteristic (ROC) curves were used to assess the diagnostic performance of clinic and angiogenic factors for identifying PE. Differences among ROC curves were tested by DeLog.

## 3 Results

### 3.1 In the Whole Cohort of Women at the Time of the Screening for GDM

Clinical and metabolic features of the 398 women included in the analysis were previously reported [14]. Among this cohort, GDM was diagnosed in 213 (53.5%) women. Data for PE (*n* = 18, 5%) were available in 361 women. GDM (67%) was more common in women with PE.

At least one sample for the angiogenic factors assay was available in all women at 24–28, 28–32 and/or 35–37 gestational weeks. There was no difference in sFlt1, PlGF levels and sFlt1/PlGF ratio between women with GDM and those with normal glucose tolerance (NGT) at the time of GDM screening, while women who developed PE had lower PlGF levels and higher sFlt1/PlGF ratio already at the time of the screening (Table 1).

**TABLE 1:** Angiogenetic factors at the time of the screening 75‐gr OGTT, in women with gestational diabetes (GDM) compared to those with normal glucose tolerance (NGT), and in women developing preeclampsia (PE) or not.

	All women (*n* = 398)	GDM women (*n* = 213)	NGT women (*n* = 185)	*p*‐value^a^	PE (*n* = 18)	No PE (*n* = 343)	*p*‐value^b^
sFlt1 (pg/mL)	1408 [986–2065]	1483 [1164–2136]	1392 [960–2059]	0.264	1557 [817–2031]	1440 [1011–2150]	0.763
PlGF (pg/mL)	475 [323–709]	449 [261–860]	478 [332–709]	0.765	218 [120–246]	483 [342–746]	**<** **0.001**
sFlt1/PlGF	3 [2.0–4.7]	3.1 [2.4–6.1]	2.9 [2.0–4.7]	0.290	6.5 [3.6–19.4]	3.0 [2.0–4.5]	**0.008**

*Note:* Values in bold indicate results with statistically significant differences.

^a^ Comparison between GDM and NGT.

^b^ Comparison between women developing PE or not.

Specifically, at screening time (24–28 gestational weeks) and at the univariate analysis, the PlGF levels and sFlt1/PlGF ratio were inversely and directly associated, respectively, with an increased risk of PE. Likewise, pre‐gestational BMI, and systolic and diastolic blood pressure measured at GDM screening, increased the risk of PE. On a multivariate analysis, PlGF and sFlt1/PlGF at 24–28 gestational weeks were associated with PE, regardless of all other clinical data (Table 2). Similar results were obtained when PlGF was included in the multivariate analysis instead of sFlt1/PlGF ratios (data not shown).

**TABLE 2:** Univariate and multivariate analysis of risk factors for pre‐eclampsia at the screening for gestational diabetes (GDM).

	Univariate analysis		Multivariate analysis	
	OR (C.I. 95%)	*p*‐value	OR (C.I. 95%)	*p*‐value
sFlt1 24–28 w	1.000 [0.999–1.001]	0.782		
PlGF 24–28 w	0.989 [0.982–0.996]	**0.003**	^a^	^a^
sFlt1/PlGF 24–28 w	1.249 [1.105–1.412]	**<** **0.001**	1.239 [1.077–1.426]	**0.003**
Age	0.987 [0.905–1.076]	0.764		
GDM	1.621 [0.595–4.419]	0.345		
Systolic blood pressure	1.051 [1.013–1.090]	**0.008**	1.024 [0.945–1.110]	0.556
Diastolic blood pressure	1.080 [1.020–1.144]	**0.009**	1.022 [0.909–1.150]	0.712
Pre‐gestational BMI	1.095 [1.013–1.183]	**0.022**	1.180 [0.932–1.494]	0.170

*Note:* Values in bold indicate results with statistically significant differences.

^a^ due to the interdependency between PlGF and sFlt1/PlGF, for the multivariate analysis , only sFlt1/PlGF was considered, which was the most associated with the risk of preeclampsia at univariate analysis.

### 3.2 Women With Gestational Diabetes Across Pregnancy

**FIGURE 1:**
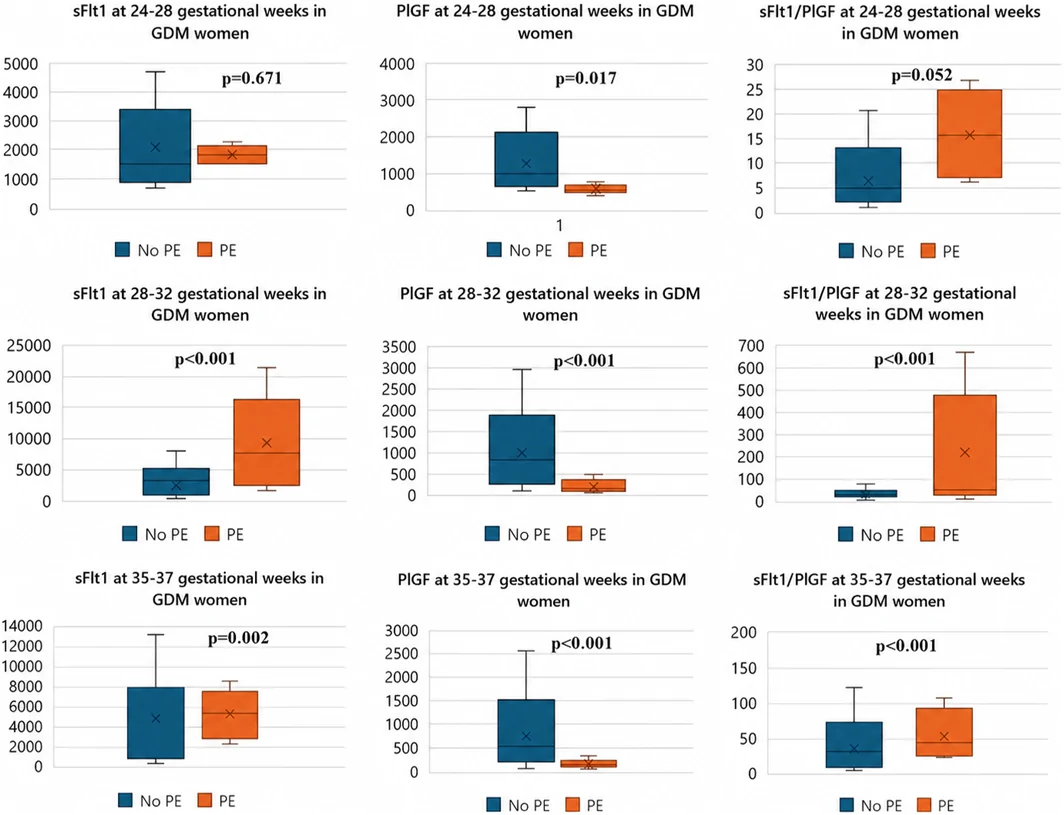
Levels of sFlt1 (pg/mL), PlGF (pg/mL) and sFlt1/PlGF ratio at 24–28, 28–32 and 35–37 gestational weeks in women with gestational diabetes (GDM) developing preeclampsia (PE) or not.

Women with GDM who developed PE had significantly lower levels of PlGF at 24–28, 28–32 and 35–37 gestational weeks, and higher sFlt1 levels and sFlt1/PlGF ratio at 28–32 and 35–37 gestational weeks compared with GDM women who did not develop PE (Figure 1). At univariate analysis, history of PE, systolic and diastolic blood pressure at GDM screening, and levels of triglycerides in the third trimester of pregnancy were all associated with an increased risk of PE. Moreover, the sFlt1/PlGF ratio at 24–28, 28–32 and 35–37 gestational weeks, and sFlt1 and PlGF levels at 28–32 and 35–37 gestational weeks were also associated with the risk of PE. The association between angiogenic factors and PE was maintained by multivariate analysis, irrespective of other clinical and metabolic data (Table 3).

**TABLE 3:** Univariate and multivariate analysis of risk factors for pre‐eclampsia in women with gestational diabetes (GDM).

	Univariate analysis		Multivariate analysis (first model)		Multivariate analysis (second model)	
	OR (C.I. 95%)	*p*‐value	OR (C.I. 95%)	*p*‐value	OR (C.I. 95%)	*p*‐value
sFlt1 24–28 w	1.000 [0.998–1.002]	0.939				
PlGF 24–28 w	0.983 [0.964–1.003]	0.095				
sFlt1/PlGF 24–28 w	1.218 [1.008–1.472]	**0.041**	^a^	^a^	^a^	^a^
sFlt1 28–32 w	1.001 [1.000–1.001]	**0.001**	^a^	^a^	^a^	^a^
PlGF 28–32 w	0.984 [0.975–0.992]	**<** **0.001**	^a^	^a^	^a^	^a^
sFlt1/PlGF 28–32 w	1.141 [1.058–1.230]	**0.001**	1.143 [1.033–1.266]	**0.010**	^a^	^a^
sFlt1 35–37 w	1.000 [1.000–1.001]	**0.007**	^a^	^a^	^a^	^a^
PlGF 35–37 w	0.978 [0.961–0.994]	**0.009**	^a^	^a^	^a^	^a^
sFlt1/PlGF 35–37 w	1.046 [1.019–1.074]	**0.001**	^a^	^a^	1.093 [1.024–1.168]	**0.008**
Age	0.930 [0.826–1.047]	0.230				
Systolic blood pressure	1.047 [1.003–1.093]	**0.036**	1.027 [0.942–1.119]	0.550	1.003 [0.916–1.099]	0.943
Diastolic blood pressure	1.106 [1.021–1.198]	**0.013**	1.015 [0.873–1.179]	0.849	1.137 [0.905–1.427]	0.269
Pre‐gestational BMI	1.087 [0.991–1.193]	0.077				
Previous PE	6.815 [1.203–38.615]	**0.030**	7.880 [0.465–133.644]	0.153	1.363 [0.018–102.277]	0.888
HbA1c	0.262 [0.027–2.514]	0.246				
Triglycerides	1.012 [1.003–1.022]	**0.012**	1.002 [0.986–1.018]	0.822	1.021 [0.988–1.056]	0.216

*Note:* Values in bold indicate results with statistically significant differences.

^a^ due to the interdependency among sFlt1, PlGF and sFlt1/PlGF, for the multivariate analysis was considered only sFlt1/PlGF., with two models (sFlt1/PlGF at 28–32 or 35–37 weeks), based on the gestational weeks of preeclampsia diagnosis. sFlt1/PlGF at 24‐28 gestational weeks was not considered, since no women developed PE in the following 4 weeks.

The sFlt1/PlGF ratio at 24–28 gestational weeks was < 38 in all women and no one developed PE in the subsequent 4 weeks. Therefore, only the sFlt1/PlGF ratios at 28–32 and 35–37 gestational weeks were used to validate the cut‐off value of 38 in women with GDM. At 28–32 gestational weeks, a sFlt1/PlGF ratio > 38 had good sensitivity (100%) and specificity (67%) to predict the risk of PE in the following 4 weeks in women with GDM, although this cut‐off value lost sensitivity (71%) and specificity (17%) at 35–37 gestational weeks.

**FIGURE 2:**
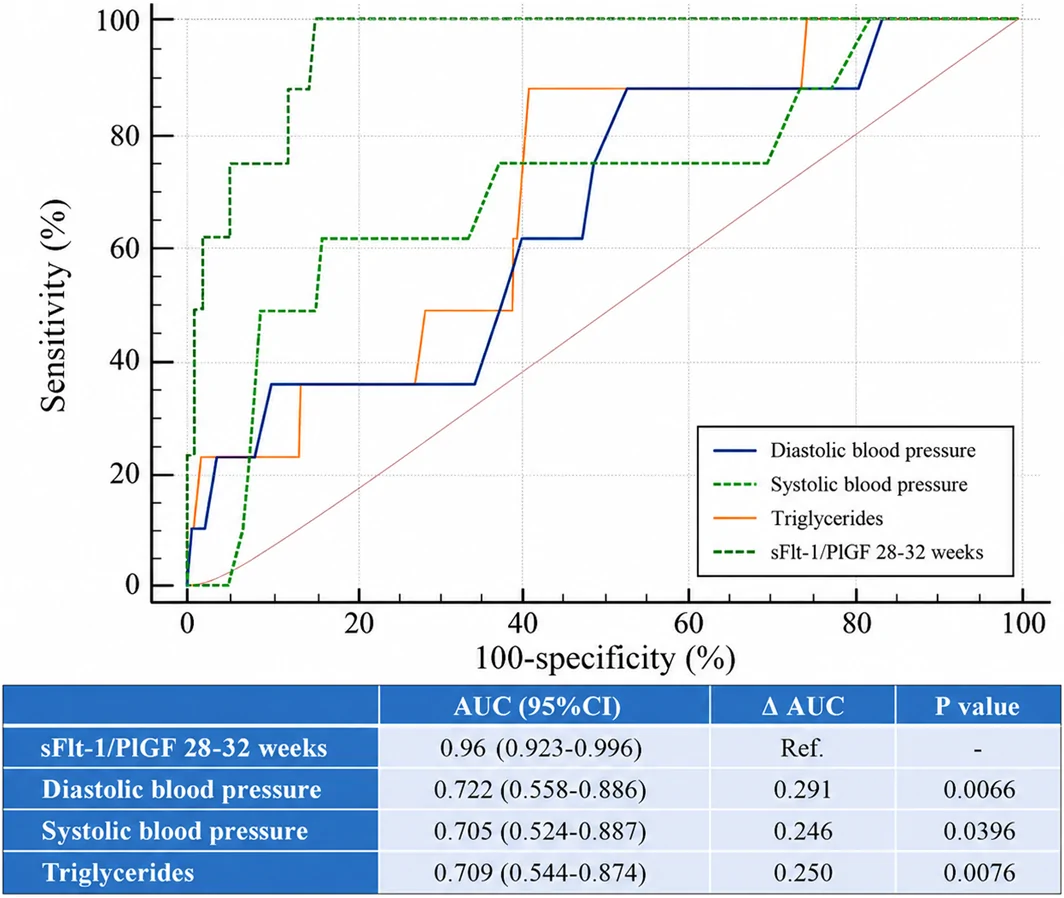
Comparison among ROC analysis of sFlt1/PlGF ratio at 28–32 gestational weeks, systolic and diastolic blood pressure and triglycerides in identifying preeclampsia (PE) in women with gestational diabetes (GDM).

The ROC analysis revealed a good performance of the sFlt1/PlGF ratio at 28–32 gestational weeks (AUC 0.960; *p* < 0.0001), systolic (AUC 0.705; *p* = 0.029) and diastolic (AUC 0.722; *p* = 0.018) blood pressure, and triglycerides (AUC 0.709; *P* = 0.045) in predicting PE. However, the sFlt1/PlGF ratio was significantly superior to clinical parameters for identifying women at risk of PE (Figure 2).

## 4 Discussion

Our study supports the potential role of PlGF measurement and sFlt1/PlGF ratio calculation at the time of GDM screening for prediction of the risk of PE in women at high risk for GDM. We found that the PlGF levels and the sFlt1/PlGF ratio were inversely and directly associated, respectively, with an increased risk of PE, irrespective of all other clinical and metabolic factors, including the GDM diagnosis. Moreover, in women with GDM, these findings suggest the superior clinical utility of the sFlt1/PlGF ratio at 28–32 gestational weeks in identifying women at PE risk.

Our findings are overall in line with those reported by others, showing significant differences in PlGF, sFlt1 levels and sFlt1/PlGF ratio at 30–34 gestational weeks in women who subsequently developed PE, irrespective of GDM diagnosis [21]. Only a few previous small studies have evaluated the relationship between angiogenic factors and PE in GDM. Specifically, in the Lara‐Barea et al. study [22] of 149 women without hypertension diagnosed in the third trimester with GDM, the sFlt‐1/PIGF ratio measured at 28–32 weeks of pregnancy was associated with a 2.70‐fold increase in the odds of developing PE (95% CI: 1.24–5.86; *p* = 0.012). Nuzzo's study [21] also highlights an association between the sFlt1/PlGF ratio and PE in women with GDM. This study also observed an increased concentration of sFlt1 in women who developed preeclampsia, although the women were not matched for gestational age to control pregnancies. At variance with this study, we did not find a difference in sFlt1 levels. The different time periods in which placental angiogenic factors were measured could explain the different relationship between the factors examined and the risk of PE. sFlt‐1 concentrations in physiological pregnancies remain constant until the 33–36 weeks of gestation, and then increase until labour and delivery [23]. Conversely, PlGF concentrations increase during the first two trimesters, reaching a peak between the 29–32 weeks and decreasing thereafter [23]. Therefore, the lack of correlation in our study between sFlt‐1 concentration and PE at the first assessment (24–28 weeks) is likely attributable to the later physiological increase of sFlt‐1 compared with PlGF. In line with these results, we found that, in women with GDM, who performed additional measurements of placental angiogenic factors in the third trimester of pregnancy, both sFlt1 and PlGF and their ratio at 28–32 and 35–37 gestational weeks were associated with increased risk of PE. This may also reflect the chronic exposure to hyperglycemia, which becomes more evident with the continuation of the pregnancy. In PE, placental ischaemia and oxidative stress result in the secretion of large amounts of anti‐angiogenic factors from the placenta, thus sustaining inflammation and endothelial dysfunction [12, 24]. Oxidative stress, in particular, increases the production of reactive oxygen species, leading to a reduction in circulating nitric oxide levels, impaired vasodilation, and over expression of the anti‐angiogenic factor sFlt1, which antagonises the pro‐angiogenic effects of PlGF [23, 25]. These alterations may be favoured by hyperglycemia in women with GDM, which induces mitochondrial damage and inflammation, inhibits trophoblast migration and invasion, and promotes trophoblast autophagy [26, 27, 28]. Although more endothelial cells and blood vessels per unit of chorionic villus were found in women with GDM, the hyperglycemia‐induced alterations may hinder the remodelling of the uterine spiral arteries, which is critical for adequate placental perfusion [29].

Our study is the first to determine the validity of the sFlt1/PlGF ratio cut‐off in women with GDM. In clinical practice a cut‐off value > 38 is used to identify women to be hospitalised for suspected PE [6]. To the best of our knowledge, this value has not been validated in women with GDM, who have a higher rate of PE (5%–10%) compared to the general population (2.5%–5%) [30, 31]. Our results now provide evidence that, even in this population, a sFlt1/PlGF > 38 at 28–32 gestational weeks effectively predicts the risk of PE within the subsequent 4 weeks. Notably, our findings point to this specific period of pregnancy for optimal use of the ratio, since its performance drops at 35–37 gestational weeks. The proposed cut‐off (sFlt‐1/PlGF > 38) demonstrated high sensitivity (100%) but moderate specificity (∼67%), while in the PROGNOSIS study, a ratio greater than 38 was associated with greater specificity (83%) and lower sensitivity (70%) in identifying women who developed PE. When comparing these data, it should be remembered once again that the women enrolled in the PROGNIOSIS study [6] were selected because of suspicion of PE and therefore likely to be at higher risk. Nonetheless, it is worth underlying that other clinical parameters had a predictive value for the risk of PE. This was the case of blood pressure [14]. However, the sFlt1/PlGF ratio at 28–32 gestational weeks offers a more precise risk assessment that could justify the excess of costs. A similar observation was made in the PROGNOSIS study [6] where the sFlt‐1:PlGF ratio predicted the onset of PE within 4 weeks better than clinical markers such as hypertension or proteinuria. It should also be noted that in our study, the prediction of preeclampsia by measuring the sflt‐1/PlGF ratio occurred in asymptomatic women, contrary to previous studies. The PROGNOSIS study, for example, identified and validated a cutoff value of 38 for the sFlt‐1:PlGF ratio as a useful predictor of the short‐term presence of preeclampsia in women with clinical signs suggestive of the disease [6]. In women with GDM, the sFlt1/PlGF ratio should be calculated at 28–32 for identification of those at higher risk of developing PE, allowing to anticipate the onset of the disease and implement a closer obstetric surveillance. Therefore, in the case of women with GDM, the sFlt‐1:PlGF ratio could find application both in the screening and triage phases.

In our cohort, the prevalence of PE was lower than that observed in other studies [12, 13].This finding may be partly explained by the close monitoring that women received during pregnancy, including frequent (every 2–3 weeks) visits by a multidisciplinary team (diabetes specialist, gynaecologist, dietician, nurse), yielding early introduction of acetylsalicylic acid or heparin therapy in cases of high risk of PE.

Some limitations of our study need to be considered. These include the relatively small sample size and the low prevalence of PE. Therefore, although the present study highlights the potential role of PlGF and sFlt1/PlGF ratio in predicting PE in women with GDM, caution is advised in interpreting the data provided, pending further confirmation in larger cohort. The strengths of our study include its prospective and monocentric design, which excludes potential differences in the management of the enrolled women, and centralised dosing of angiogenic factors, which reduces the risk of dosing errors.

In conclusion, in pregnant women at high risk for GDM, determination of PlGF levels and calculation of the sFlt1/PlGF ratio at GDM screening time (24–28 gestational weeks) are inversely and directly associated with an increased risk of PE, respectively, regardless of other clinical and metabolic factors. Moreover, in women with GDM, the sFlt1/PlGF ratio at 28–32 gestational weeks may be useful for identifying those with a higher risk of PE development, who should be referred to closer obstetric surveillance. In particular, as in non‐GDM women, sFlt1/PlGF ratio > 38 may identify those who will develop PE in the following 4 weeks, better than clinical parameters.

## Author Contributions

F.C. contributed to data collection, performed statistical analysis, and wrote the original draft. C.B. designed the study, contributed to data collection, performed statistical analysis, reviewed and edited the manuscript, and is the guarantor of this work and, as such, had full access to all the data in the study and take responsibility for the integrity of the data and the accuracy of the data analysis. F.N. contributed to data collection, performed statistical analysis, and wrote the original draft. M.A. contributed to data collection and reviewed the manuscript. G.D.G. contributed to data collection and reviewed the manuscript. S.D.P. reviewed the manuscript. G.P. reviewed the manuscript. L.B. devised and designed the study, contributed to data collection, and reviewed and edited the manuscript

## Funding

This study was supported by Roche Diagnostics that provided Elecsys kits for dosing sFlt1andPlGF.

## Conflicts of Interest

The authors declare no conflicts of interest.

## Data Availability

The data that support the findings of this study are available on request from the corresponding author. The data are not publicly available due to privacy or ethical restrictions.

## References

[1] E. Abalos , C. Cuesta , A. L. Grosso , D. Chou , and L. Say , “Global and Regional Estimates of Preeclampsia and Eclampsia: A Systematic Review,” European Journal of Obstetrics & Gynecology and Reproductive Biology 170, no. 1 (2013): 1–7, 10.1016/j.ejogrb.2013.05.005. 23746796

[2] H. T. Lee‐Ann , M. A. Brown , G. J. Mangos , and G. K. Davis , “Transient Gestational Hypertension: Not Always a Benign Event,” Pregnancy Hypertension 2, no. 1 (2012): 22–27, 10.1016/j.preghy.2011.09.001. 26104985

[3] T. Chaiworapongsa , P. Chaemsaithong , L. Yeo , and R. Romero , “Pre‐Eclampsia Part 1: Current Understanding of Its Pathophysiology,” Nature Reviews Nephrology 10, no. 8 (2014): 466–480, 10.1038/nrneph.2014.102. 25003615 PMC5893150

[4] O. Erez , R. Romero , J. Espinoza , et al., “The Change in Concentrations of Angiogenic and Anti‐Angiogenic Factors in Maternal Plasma Between the First and Second Trimesters in Risk Assessment for the Subsequent Development of Preeclampsia and Small‐for‐Gestational Age,” Journal of Maternal‐Fetal and Neonatal Medicine 21, no. 5 (2008): 279–287, 10.1080/14767050802034545. 18446652 PMC2846114

[5] R. Akolekar , A. Syngelaki , L. Poon , D. Wright , and K. H. Nicolaides , “Competing Risks Model in Early Screening for Preeclampsia by Biophysical and Biochemical Markers,” Fetal Diagnosis and Therapy 33, no. 1 (2013): 8–15, 10.1159/000341264. 22906914

[6] K. Duckitt and D. Harrington , “Risk Factors for Pre‐Eclampsia at Antenatal Booking: Systematic Review of Controlled Studies,” BMJ 330, no. 7491 (2005): 565, 10.1136/bmj.38380.674340.e0. 15743856 PMC554027

[7] H. Zeisler , E. Llurba , F. Chantraine , et al., “Predictive Value of the sFlt‐1:PlGF Ratio in Women With Suspected Preeclampsia,” New England Journal of Medicine 374, no. 1 (2016): 13–22, 10.1056/NEJMoa1414838. 26735990

[8] P. R. Garner , M. E. D'Alton , D. K. Dudley , P. Huard , and M. Hardie , “Preeclampsia in Diabetic Pregnancies,” American Journal of Obstetrics and Gynecology 163, no. 2 (1990): 505–508, 10.1016/0002-9378(90)91184-e. 2386136

[9] M. J. Bond and J. G. Umans , “Microvascular Complications and the Diabetic Pregnancy,” Current Diabetes Reports 6, no. 4 (2006): 291–296, 10.1007/s11892-006-0063-2. 16879781

[10] L. Suhonen and K. Teramo , “Hypertension and Pre‐Eclampsia in Women With Gestational Glucose Intolerance,” Acta Obstetricia et Gynecologica Scandinavica 72, no. 4 (1993): 269–272, 10.3109/00016349309068036. 8389513

[11] H. S. Ros , S. Cnattingius , and L. Lipworth , “Comparison of Risk Factors for Preeclampsia and Gestational Hypertension in a Population‐Based Cohort Study,” American Journal of Epidemiology 147, no. 11 (1998): 1062–1070, 10.1093/oxfordjournals.aje.a009400. 9620050

[12] S. Rana , E. Lemoine , J. P. Granger , and S. A. Karumanchi , “Preeclampsia: Pathophysiology, Challenges, and Perspectives,” Circulation Research 124, no. 7 (2019): 1094–1112, 10.1161/circresaha.118.313276. 30920918

[13] H. D. McIntyre , P. Catalano , C. Zhang , G. Desoye , E. R. Mathiesen , and P. Damm , “Gestational Diabetes Mellitus,” Nature Reviews Disease Primers 5, no. 1 (2019): 47, 10.1038/s41572-019-0098-8. 31296866

[14] F. Nicolì , F. Citro , L. Battini , et al., “Prevalence and Predictive Risk Factors of Hypertensive Disorders in Pregnant Women at High Risk for Gestational Diabetes. The PREeclampsia in DIabetiC gestaTION (PREDICTION) Study,” Journal of Endocrinological Investigation (2025). 10.1007/s40618-024-02520-1 39883312

[15] AMD‐SID , Standard Italiani per la Cura del Diabete Mellito, (2018).

[16] B. E. Metzger , S. G. Gabbe , B. Persson , et al., “International Association of Diabetes and Pregnancy Study Groups Recommendations on the Diagnosis and Classification of Hyperglycemia in Pregnancy,” Diabetes Care 33, no. 3 (2010): 676–682, 10.2337/dc10-0719. 20190296 PMC2827530

[17] WHO Guidelines Approved by the Guidelines Review Committee , Diagnostic Criteria and Classification of Hyperglycaemia First Detected in Pregnancy (World Health Organization 2013, 2013). 24199271

[18] (AIPE) AIP , I disordini ipertensivi in gravidanza: classificazione, diagnosi e terapia. Raccomandazioni di buona pratica clinica.

[19] A. L. Tranquilli , G. Dekker , L. Magee , et al., “The Classification, Diagnosis and Management of the Hypertensive Disorders of Pregnancy: A Revised Statement From the ISSHP,” Pregnancy Hypertension 4, no. 2 (2014): 97–104, 10.1016/j.preghy.2014.02.001. 26104417

[20] National Institute for Health and Care Excellence: Guidelines , Hypertension in Pregnancy: Diagnosis and Management (National Institute for Health and Care Excellence (NICE) 2019, 2019). 31498578

[21] A. M. Nuzzo , D. Giuffrida , L. Moretti , et al., “Placental and Maternal sFlt1/PlGF Expression in Gestational Diabetes Mellitus,” Scientific Reports 11, no. 1 (2021): 2312, 10.1038/s41598-021-81785-5. 33504861 PMC7840991

[22] A. Lara‐Barea , B. Sánchez‐Lechuga , A. Campos‐Caro , et al., “Angiogenic Imbalance and Inflammatory Biomarkers in the Prediction of Hypertension as Well as Obstetric and Perinatal Complications in Women With Gestational Diabetes Mellitus,” Journal of Clinical Medicine 11, no. 6 (2022): 1514, 10.3390/jcm11061514. 35329840 PMC8953606

[23] R. J. Levine , S. E. Maynard , C. Qian , et al., “Circulating Angiogenic Factors and the Risk of Preeclampsia,” New England Journal of Medicine 350, no. 7 (2004): 672–683, 10.1056/nejmoa031884. 14764923

[24] L. C. Chappell , C. A. Cluver , J. Kingdom , and S. Tong , “Pre‐Eclampsia,” Lancet 398, no. 10297 (2021): 341–354, 10.1016/s0140-6736(20)32335-7. 34051884

[25] M. Lappas , U. Hiden , G. Desoye , J. Froehlich , S. Hauguel‐de Mouzon , and A. Jawerbaum , “The Role of Oxidative Stress in the Pathophysiology of Gestational Diabetes Mellitus,” Antioxidants and Redox Signaling 15, no. 12 (2011): 3061–3100, 10.1089/ars.2010.3765. 21675877

[26] M. Gauster , G. Desoye , M. Tötsch , and U. Hiden , “The Placenta and Gestational Diabetes Mellitus,” Current Diabetes Reports 12, no. 1 (2012): 16–23, 10.1007/s11892-011-0244-5. 22102097

[27] C. S. Han , M. A. Herrin , M. C. Pitruzzello , et al., “Glucose and Metformin Modulate Human First Trimester Trophoblast Function: A Model and Potential Therapy for Diabetes‐Associated Uteroplacental Insufficiency,” American Journal of Reproductive Immunology 73, no. 4 (2015): 362–371, 10.1111/aji.12339. 25394884 PMC4356646

[28] K. R. Heim , M. J. Mulla , J. A. Potter , C. S. Han , S. Guller , and V. M. Abrahams , “Excess Glucose Induce Trophoblast Inflammation and Limit Cell Migration Through HMGB1 Activation of Toll‐Like Receptor 4,” American Journal of Reproductive Immunology 80, no. 5 (2018): e13044, 10.1111/aji.13044. 30175447

[29] F. Troncoso , J. Acurio , K. Herlitz , et al., “Gestational Diabetes Mellitus Is Associated With Increased Pro‐Migratory Activation of Vascular Endothelial Growth Factor Receptor 2 and Reduced Expression of Vascular Endothelial Growth Factor Receptor 1,” PLoS One 12, no. 8 (2017): e0182509, 10.1371/journal.pone.0182509. 28817576 PMC5560693

[30] C. W. Redman and I. L. Sargent , “Latest Advances in Understanding Preeclampsia,” Science 308, no. 5728 (2005): 1592–1594, 10.1126/science.1111726. 15947178

[31] J. A. Hutcheon , S. Lisonkova , and K. S. Joseph , “Epidemiology of Pre‐Eclampsia and the Other Hypertensive Disorders of Pregnancy,” Best Practice & Research Clinical Obstetrics & Gynaecology 25, no. 4 (2011): 391–403, 10.1016/j.bpobgyn.2011.01.006. 21333604

